# Characterization of the Antidiabetic Role of *Parkinsonia aculeata* (Caesalpineaceae)

**DOI:** 10.1155/2011/692378

**Published:** 2010-09-19

**Authors:** Ana Catarina Rezende Leite, Tiago Gomes Araújo, Bruno de Melo Carvalho, Maria Bernadete Souza Maia, Vera Lúcia de Menezes Lima

**Affiliations:** ^1^Departamento de Bioquímica, Centro de Ciências Biológicas, Universidade Federal de Pernambuco, Cidade Universitária, 50670-901, Recife, PE, Brazil; ^2^Departamento de Fisiologia e Farmacologia, Centro de Ciências Biológicas, Universidade Federal de Pernambuco, Recife, PE, Brazil

## Abstract

This paper reports the characterization of the antidiabetic role of a hydroethanolic extract from *Parkinsonia* aerial parts (HEPA), in normal and alloxan-induced diabetic rats, treated with HEPA (125 and 250 mg/kg; p.o.). Oral glucose tolerance test, acute oral toxicity test and preliminary phytochemical analyses were performed. The diabetic rats treated with HEPA showed a significant reduction in serum and urinary glucose, urinary urea and triglyceride levels, as compared to the diabetic untreated group. However, in the normal treated groups, a significant reduction was found only in serum triglyceride levels. In all treated diabetic groups, an improvement in hepatic glycogen was observed, as well as a decrease in liquid intake and urinary volume, and an enhancement in the weight of skeletal muscles (*soleus* and *extensor digitorum longus*), kidneys and epididymal adipose tissue. Nevertheless, body and liver weights were ameliorated only in the diabetic group treated with HEPA (250 mg/kg). Moreover, oral glucose tolerance was higher in animals treated with HEPA, while results also showed that HEPA could be considered toxicologically safe. Phytochemical analysis revealed the presence of tanins, flavonoids and steroids in HEPA. In conclusion, *P. aculeata* presents an antidiabetic activity and other beneficial effects that ameliorate diabetes and associated complications.

## 1. Introduction

The World Health Organization (WHO) estimates that more than 180 million people worldwide have diabetes and that this number is projected to double by year 2030 when this disorder will be affecting people, irrespective of sex, age, and socioeconomic status [1, 2]. Currently, conventional drugs used for diabetes treatment are associated with drawbacks such as rigid dosing regimens, highcost, inaccessibility, and unexpected side effects [3, 4]. Therefore, screening for new antidiabetic compounds from natural plants used in folk medicine is still attractive because of their efficacy, low incidence of side effects, and low cost [5, 6]. Ethnobotanical reports indicate that over 1200 species of plants have been reported as traditional medicines for diabetes [7]. In Brazil, medicinal plants are used according to native folk traditions, or according to the traditions of worldwide immigrants that use these plants for formulation of home remedies such as teas, decoctions or tinctures. Around 60% of the population make use of these kinds of agents because of limited access to health services and lack of financial resources [8]. However, in order to explore the efficacy, mechanism of action, and safety of natural products, there is need to perform preclinical and clinical experiments. WHO suggests the evaluation of potential effective therapeutic plants, especially in areas where safe and modern drugs are not available [4, 9].

*Parkinsonia aculeata* L. (Caesalpineaceae) is a medium-sized tree (4–6 m) found in the Xingó region (semiarid area) in the Northeast of Brazil, used as traditional medicine by the local population and traditional healers for the empiric treatment of hyperglycemia, without scientific background [10]. In addition to these properties, *P. aculeata* has a wide range of pharmacological and biological activities, including antimalarial and antimicrobial properties [11, 12]. 

A previous study performed by our group demonstrated the antihyperglycemic and antihyperlipidemic activities of an aqueous extract fraction of *P. aculeata* aerial parts in alloxan-induced diabetic rats [13]. However, studies of this plant are rarely reported in the literature. Since, the aqueous extract fraction of *P. aculeata* was found to have a high antidiabetic potential, the study of its hydroethanolic extract was therefore undertaken to further investigate its antidiabetic actions in alloxan-induced diabetic as well as in normal rats. Antidiabetic effects were evaluated by measuring a spectrum of diabetes-related parameters, such as oral glucose tolerance and physiological and biochemical parameters related to carbohydrate, lipid, and protein metabolism. Additionally, tests for acute oral toxicity and preliminary phytochemical analyses were also carried out.

## 2. Methods

### 2.1. Plant Material

Aerial parts of *P. aculeata* were collected from the Xingó region (Sergipe, Brazil), during May 2007. The plant was identified by Professor H. P. Bautista (INCRA-BA) and a voucher specimen was deposited (n° 500) in the Xingó Herbarium (Canindé do São Francisco, Sergipe, Northeast region, Brazil).

### 2.2. Preparation of Plant Extract

Dehydrated and powdered *P. aculeata* aerial parts (50 g) were macerated with EtOH: H_2_O (1 : 1; v/v). The suspension was submitted to mechanic agitation for one hour at 23°C and subsequently placed in a refrigerator for 24 h and then filtered through cotton wool. After filtration, the material was lyophilized and stored at −20°C until use. The final yield of the hydroethanolic extract of *P. aculeata *aerial parts (HEPA) was 7 g/50 g of the dried powdered aerial parts. For pharmacological assays, a fresh dilution of lyophilized hydroethanolic extract in vehicle (distilled water) was prepared on the day of treatment and administered by gavage at doses of 125 and 250 mg/kg b.w. in a fixed volume of 1 mL.

### 2.3. Preliminary Phytochemical Screening

A simple qualitative and semiquantitative phytochemical analysis was carried out on HEPA for the identification of various major classes of active chemical constituents: alkaloids, saponins, tanins, flavonoids, and steroids, as described previously in [14, 15].

### 2.4. Animals

The study was carried out with adult male Wistar rats weighing 180–250 g. Animals were acclimatized to the experimental conditions in metabolic cages and kept under standard environmental conditions (22 ± 3°C; 12/12 h light/dark cycle). Rats were allowed to feed on chow (Labina Purina—Brazil, CO) and water *ad libitum*. The animals were adapted to metabolic cages 3 days prior to the experiment. For acute toxicity test, adult male Swiss mice (30–35 g body weight) were used and kept under the same environmental conditions. All experiments reported herein are in accordance with the Animal Care and Use Committee at the Federal University of Pernambuco and guidelines for Care and Use of Laboratory Animals.

### 2.5. Acute Oral Toxicity Test

Acute toxicity (LD_50_) was estimated in mice using the dose limit of the up and down procedure, according to the Organization for Economic Cooperation and Development (OECD) test guideline 425. Acute Oral Toxicity was estimated using a computer-based Statistical Program—AOT425statPgm, version: 1.0, at a dose limit of 5 g/kg body weight/oral route and default of Sigma at 0.5 [16, 17].

### 2.6. Induction of Diabetes

Diabetes was induced in overnight-fasted rats by a single intraperitoneal injection of 150 mg/kg b.w. of freshly-prepared alloxan monohydrate (Sigma, St. Louis, MO, USA), dissolved in sterile saline solution (0.9% NaCl). After alloxan administration, all animals were relocated to their cages and given free access to food and water. The diabetic state was assessed by measuring the serum blood glucose levels 3 days afterwards. Rats with fasting glucose of above 250 mg/dL were considered diabetic.

### 2.7. Evaluation of the Subchronic Oral Treatment

The rats were divided into seven groups (3 normal and 4 diabetic), consisting of 6 animals each. For normal rats, the control group was assigned to normal control (NC), which received the vehicle, and two groups of normal treated rats that received HEPA by gavage daily at doses of 125 mg/kg (NT125) or 250 mg/kg (NT250). The groups of diabetic rats were as follows. Diabetic untreated group, which received the vehicle and was assigned as the diabetic control group (DC), and two groups of diabetic-treated rats that received HEPA by gavage daily at doses of 125 mg/kg (DT125) or 250 mg/kg (DT250). Additionally, another group of diabetic rats was treated with insulin (DTI) and adopted as a positive treatment group. This group was treated twice a day (8 a.m. and 5 p.m.) by subcutaneous injection with 3 units of NPH insulin (Humolin NU-100, Eli Lilly, Brazil). All treatments were carried out for 16 days.

### 2.8. Oral Glucose Tolerance Test

Oral glucose tolerance test was performed using a method previously described by Sezik and coworkers [18]. The animals were randomly divided into six groups (*n* = 6 animal/group), as follows: DC, DT125, DT250, NC, NT125, and NT250. After overnight fasting, the 0-min blood sample was taken from the rats by orbital sinus puncture for determination of fasting blood glucose. Glucose (2 g/kg b.w.) was orally administered at 30 min after an oral administration of the plant extract or vehicle (for control). Blood glucose was measured by enzymatic methods, just before and 1/2 h, 1 h, 2 h, 4 h after the oral administration of the HEPA.

### 2.9. Biochemical and Physiological Analysis

Under light ether anesthesia, blood samples were withdrawn from the rat-orbital sinus with a capillary tube for biochemical parameters determination. Serum glucose, cholesterol, triglycerides, HDL-cholesterol, urinary glucose, and urea were measured by enzymatic methods (Labtest Diagnostica, Brazil/SA). Hepatic glycogen was extracted with 30% KOH and precipitated with alcohol [19] and quantified by the colorimetric anthrone method [20]. In addition, body weight, food and liquid intake (water), and urine volume were also evaluated. All the parameters were measured before and on the 16th day of treatment. After the last measurement, the rats were sacrificed by decapitation. The epididymal adipose tissue (EAT) lying over psoas, the *soleus *and *extensor digitorum longus *(EDL) skeletal muscles, kidney, and liver were removed and weighed.

### 2.10. Statistical Analysis

The results of experiments performed in at least six independent experiments in duplicate, are displayed as mean ± S.E.M. Multiple comparisons were tested by one-way ANOVA, followed by Tukey's *post hoc* test, with the significance level set at *P* < .05 using SPSS software (SPSS for Windows, version 16.0, Chicago, IL).

## 3. Results

### 3.1. Phytochemical Analysis

The synopsis of the simple qualitative and quantitative analysis carried out on the plant extract is shown in Table 1.

### 3.2. Acute Oral Toxicity Test

Oral administration of a dose limit of 5 g/kg of the HEPA did not result in any lethality or observable behavioral changes such as writhing, gasping, palpitation, and decreased respiratory rate, in the treated mice. The oral acute toxicity of *P. aculeata* was, therefore, considered as unclassified, since the LD_50_ estimate from the AOT425Pgm program was greater than 5 g/kg of body weight/oral route.

### 3.3. Impact of HEPA on Fasting Serum Glucose and Urinary Glucose Levels

**Figure 1:**
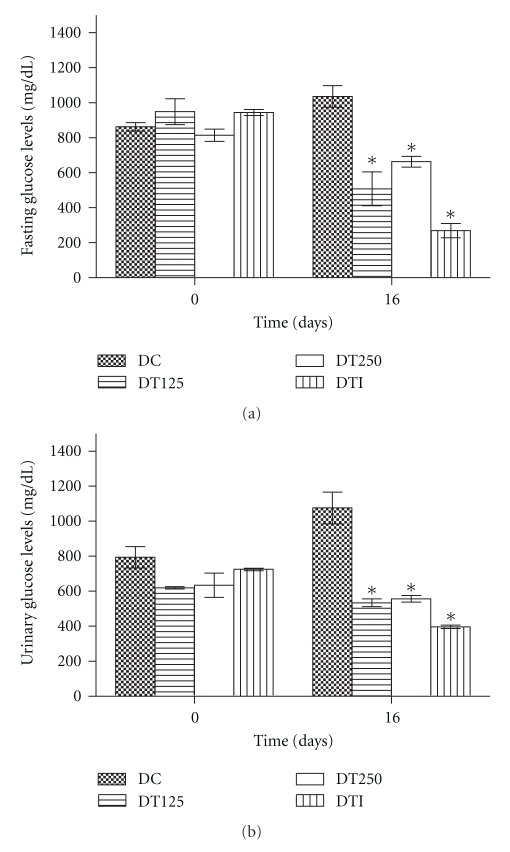
*Fasting serum glucose (a) and urinary glucose (b) levels in diabetic rats after subchronic treatment with HEPA.* DC: diabetic control (vehicle); DT125: diabetic-treated with HEPA (125 mg/Kg); DT250: diabetic treated with HEPA (250 mg/Kg); DTI: diabetic treated with insulin. All values expressed as mean ± S.E.M. (*n* = 6). One-way ANOVA with Tukey's *post hoc* test was applied. *P < .05 versus DC group.

Daily treatment for 16 days with HEPA (125 and 250 mg/kg; p.o.), in alloxan-induced diabetic rats, significantly reduced serum glucose levels (Figure 1(a)) in DT125 (51%), DT250 (36%), and DTI (74%) groups, when compared to the DC group.  Figure 1(b) shows that the treatment with HEPA for the same period also significantly decreased urinary glucose levels in diabetic-treated groups. Once again, the positive control (DTI) decreased urinary glucose levels. On the other hand, comparison of normal (nondiabetic) groups (NC, NT125 and NT250) during the entire 16 days of treatment showed no overall significant differences among these groups, regarding serum glucose and urinary glucose levels (data not shown).

### 3.4. HEPA Ameliorates Liver Glycogen Content in Diabetic Rats

**Figure 2:**
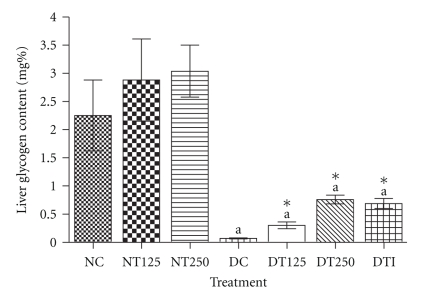
*Hepatic glycogen content from diabetic and normal rats after subchronic treatment with HEPA.* NC: nondiabetic control (vehicle); NT125: nondiabetic treated with HEPA (125 mg/Kg); NT250: nondiabetic treated with HEPA (250 mg/Kg); DC: diabetic control (vehicle); DT125: diabetic treated with HEPA (125 mg/Kg); DT250: diabetic treated with HEPA (250 mg/Kg); DTI: diabetic treated with insulin. All values represent mean ± S.E.M. (*n* = 6). One-way ANOVA with Tukey's *post hoc* test. ^a^ *P* < .001 versus NC; *P < .01 versus DC.

Figure 2 demonstrates that, as expected, the alloxan-diabetic rats presented a significant decrease in hepatic glycogen content, when compared with nondiabetic rats. However, a significant increase was observed in hepatic glycogen levels in diabetic rats after the subchronic HEPA treatment with the concentrations of 125 mg/kg (4.3-fold) and 250 mg/kg (10.8-fold), which showed similar effects when compared with the usual drug of treatment, insulin (9.8-fold). In addition, we observed that this parameter was increased with no statistical significance (*P* > .05) in normal rats treated with HEPA.

### 3.5. Serum Triglycerides, Total Cholesterol and HDL-Cholesterol Levels on HEPA Administration

The effect of oral administration of HEPA on serum triglycerides, total cholesterol, and HDL-cholesterol levels are displayed in Table 2. In comparison with the control values (DC), HEPA at the dose of 250 mg/kg b.w. (DT250) exhibited a reduction in serum triglyceride levels (74%) that was more pronounced than that observed with 125 mg/kg b.w. (DT125) (69%) and that produced by insulin treatment (48%). Moreover, the normal treated groups (NT125 and NT250), in comparison with the NC group, showed significant reductions in serum triglyceride levels of 19% and 31%, respectively. Total cholesterol and HDL-cholesterol serum levels did not suffer any significant alteration in any of the groups (normal and diabetic rats) subjected to the subchronic HEPA treatment.

### 3.6. HEPA Reduces Urinary Urea Levels in Diabetic Rats

**Figure 3:**
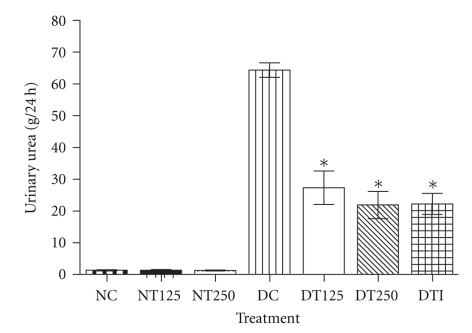
*Urinary urea level from animals after 16 days of treatment with HEPA. *NC: nondiabetic control (vehicle); NT125: nondiabetic treated with HEPA (125 mg/Kg); NT250: nondiabetic treated with HEPA (250 mg/Kg); DC: diabetic control (vehicle); DT125: diabetic treated with HEPA (125 mg/Kg); DT250: diabetic treated with HEPA (250 mg/Kg); DTI: diabetic treated with insulin. The values represent the mean ± S.E.M. (*n* = 6). One-way ANOVA with Tukey's *post hoc* test. *P < .05 versus DC.

The subchronic treatment with HEPA in the DT125 and DT250 groups resulted in a significant decrease in urinary urea levels (g/24 h) (Figure 3). The reductions observed in the DT125 (58%) and DT250 (66%) groups were similar to that obtained in the DTI group (65%). In the normal treated groups, the urinary urea concentration was unaffected.

### 3.7. HEPA Improves Some Physiological Variables in Diabetic Rats

The effects of HEPA on physiological variables (body weight, liquid and food intake, and urinary volume) are displayed in Table 3 for diabetic groups. A significant increase in body weight was observed in the DT250 and DTI groups when compared with the DC group. Indeed, the DT125, DT250, and DTI groups showed significant decreases in liquid intake and urinary volume. No change was observed in food intake in the diabetic groups. Additionally, HEPA treatment did not modify the physiological variables cited in nondiabetic rats (data not shown).

### 3.8. Effect of HEPA on Liver, Kidney, EAT, EDL, and Soleus Weight

As shown in Table 3, the treatment with HEPA produced an improvement in kidneys, EAT, and skeletal muscles (*soleus* and EDL) weight in diabetic-treated rats, when compared to DC. Furthermore, the liver weight was improved only in the DT250 group, compared to DC. Additionally, it may be observed that HEPA treatment has no apparent effect on normal rats (data not shown).

### 3.9. Oral Glucose Tolerance Test (OGTT)

**Figure 4:**
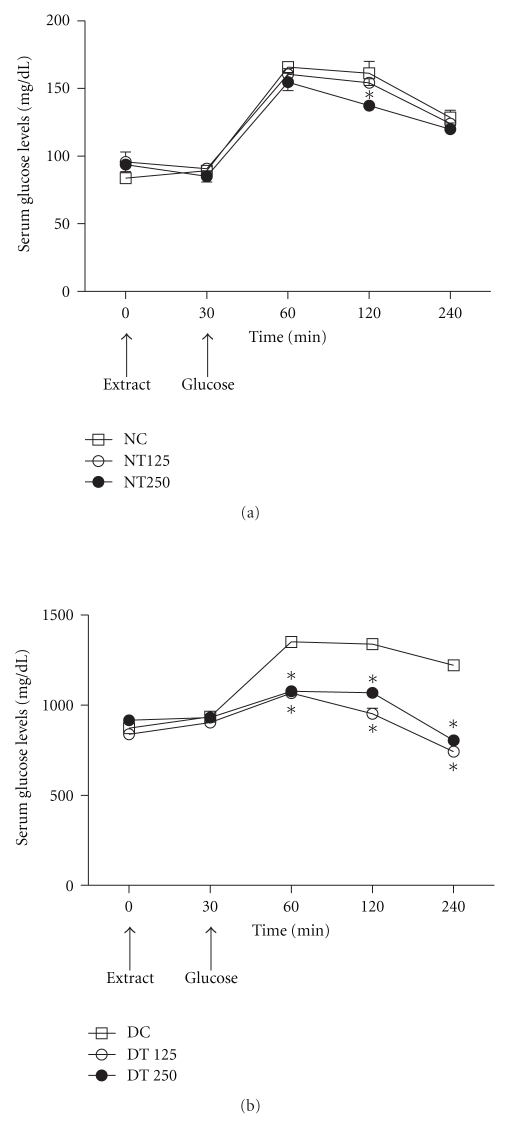
*Oral glucose tolerance test (OGTT) in normal (a) and diabetic (b) rats treated with HEPA.* NC: nondiabetic control (vehicle); NT125: nondiabetic treated with HEPA (125 mg/Kg); NT250: nondiabetic treated with HEPA (250 mg/Kg); DC: diabetic control (vehicle); DT125: diabetic treated with HEPA (125 mg/Kg); DT250: diabetic treated with HEPA (250 mg/Kg). Each value is expressed as the mean ± S.E.M. (*n* = 6). For statistical analysis was employing one-way ANOVA with Tukey's *post hoc* test. *P < .05 versus respective control group (NC or DC). Arrows indicate the time of administration of extract or glucose.

In diabetic rats, the administration of HEPA (125 and 250 mg/kg b.w.) orally at 30 min prior to glucose load resulted in an improvement in glucose tolerance (Figure 4(b)). As shown, in all diabetic and normal groups the serum glucose concentration peak was observed after 1 h. DT125 and DT250 treated rats demonstrated improvements in glucose tolerance of 21% and 20%, 28% and 20%, 39% and 34% at 1, 2, and 4 h, respectively, in comparison with the DC group. Conversely in normal rats, only the NT250-treated group displayed a slightly improved glucose tolerance at 2 h, when compared with normal control rats (NC) (Figure 4(a)).

## 4. Discussion

The present study investigated the antidiabetic effects of the hydroethanolic extract of *P. aculeata* in a model of alloxan-induced diabetic rats as well as in normal rats. Alloxan causes diabetes through its ability to generate reactive oxygen species (ROS), which leads to a massive increase in cytosolic calcium concentration and, consequently, a rapid destruction in the insulin-producing beta cells of the pancreas [21, 22]. In the experimental model adopted in this work, the animals submitted to alloxan injection presented clear symptoms of severe diabetes, such as hyperglycemia, glucose intolerance, muscular proteolysis, and adipose tissue lipolysis leading to body weight loss, glycosuria, osmotic polyuria, and other consequences. As expected, the improvement in all assessed parameters produced by insulin treatment validates our model.

Results found in this study indicate that the hydroethanolic extract from *P. aculeata* (HEPA, 125 and 250 mg/kg), administered orally for 16 days, is able to produce significant beneficial effects on carbohydrate metabolism in diabetic rats, by reducing both serum and urinary glucose in treated animals (Figures 1(a) and 1(b)). In addition, HEPA had no effect on carbohydrate metabolism in normal rats (data not shown).

Liver glycogen is the primary storable form of glucose and its levels are a direct reflection of insulin signaling [23]. Consequently, in diabetes mellitus, a marked decrease in liver glycogen levels is observed [24, 25]. Accordingly, our data also showed significant decrease in liver glycogen content in diabetic rats; however, these animals presented an increase in liver glycogen content after the treatment with HEPA (Figure 2). This effect could be attributed to stimulation of glycogenesis and/or inhibition of glycogenolysis, possibly linked to improvement on insulin signaling. This effect represents a significant contribution to glycemic control. As reported in this paper, improvements in other related diabetic parameters, such as decreased osmotic diureses and a reduction in water intake (Table 3) were predictable as an outcome of decreased glycemia. In accordance to our results, other species of the Caesalpineaceae family, popularly used as antidiabetics, have also been showed similar effects on carbohydrate metabolism [26–29]. 

In diabetes, there is an increase in protein catabolism, mainly in skeletal muscles, with the flow of amino acids into the liver, providing substrates to gluconeogenesis [30]. In this way, changes in the proteolytic pathways promoted by insulin deficiency lead to a marked muscular proteolysis, responsible for muscular mass decrease [31]. Our reports have shown that HEPA treatment in diabetic rats is able to reduce the levels of urinary urea (Figure 3) as well as cause an improvement in the weight of skeletal muscles (*soleus* and EDL) (Table 3). These findings might indicate that HEPA exerts its effect by the inhibition of gluconeogenesis. In addition, although the diabetic control group presented a body weight reduction, weight loss was lower in the diabetic-treated groups. These beneficial effects could be related to the ability to repair the protein breakdown and enhance the glycemic control produced by the HEPA treatment.

Dysregulation of the lipid profile is one of the most common complications in diabetes and found in 40% of diabetic cases [32]. Alloxan-induced diabetic rats showed an important lipolytic activity, due to the insulinopenic state that contributes to this alteration in the lipid profile [33]. As shown in this work, the HEPA treatment did not present any improvement in cholesterol and HDL—cholesterol levels in diabetic- as well as normal-treated rats (Table 2); these results differ from those of our previous study that employed the aqueous extract fraction [13]. Conversely, HEPA treatment presented a relevant hypotriglyceridemic effect in diabetic rats and, surprisingly, this effect was also observed in normal rats (Table 2). These consequences of HEPA treatment on the lipid profile support a favorable effect on EAT weight of diabetic rats and suggest that the mobilization of fatty acids from the peripheral fat depots could be reduced. Recently, Sharma and co-workers [34] demonstrated that *P. aculeata* seeds contain a high amount of polyunsaturated fatty acids (PUFA). Several studies have demonstrated that the intake of PUFA is associated with a decrease in risk factors, such as triglycerides levels, in cardiovascular disease [35, 36]. Thus, these new data, reported for *P. aculeata* is in line with the observed hypotriglyceridemic effect.

In this study, we also observed effects of the treatment with HEPA on the liver and kidney weights of diabetic-treated rats (Table 3). Only the DT250-treated group showed a significant reduction in liver weight. The literature regarding the effect of diabetes on liver weight is contradictory, as some studies have shown an increase in hepatic weight [37], while others report no change [38]. With regard to kidney weight, the treatment with HEPA caused a significant reduction. Diabetic animals tend to show renal hypertrophy caused by an increased formation of advanced glycation end products (AGE) and accumulation of glycogen granules in distal tubules [39, 40].

In our previous study, it was reported that the aqueous extract fraction of *P. aculeata* has antidiabetic properties [13]. The current study shows that the hydroethanolic extract of *P. aculeata* also has antidiabetic effects, but shows less potent effects than the aqueous extract fraction. The antidiabetic plant extracts may involve one or more compounds to produce its effects, suggesting that the natural constituents could act separately or synergistically [7]. Our preliminary phytochemical screening of HEPA showed that it contains tannins, steroids, and a high concentration of flavonoids. Additionally, previous phytochemical studies of the *P. aculeata* leaves extract have shown the presence of orientin, isoorientin, vitexin, and isovitexin (all glycosylated flavonoids) [41–43], which have beneficial effects in diabetes, ameliorating glucose tolerance, lipid profile, glycogen biosynthesis, glucose uptake, and insulin signaling [44–46]. In accordance with such observations, Sezik et al. [18] suggested that the potent antidiabetic activity of isoorientin is associated with its specific interaction with pancreatic *β*-cells after systemic absorption and subsequent reversion of the oxidative injury promoted by alloxan. In this context, it is probable that the glycosylated flavonoids found in the *P. aculeata* extract produce its antidiabetic properties. These compounds have a hydrosoluble carbohydrate radical that is better extracted by more polar solvents, possibly explaining the stronger antidiabetic effects presented by treatment with the aqueous extract fraction of *P. aculeata*.

One goal of therapy for diabetic patients is the maintenance of normal blood glucose levels, including control of postprandial increases in blood glucose [47]. The glucose load was well tolerated in the extract-treated groups. Moreover, only the NT250 normal treated group presented a discrete improvement in glucose tolerance (Figure 4(a)). However, this effect was not sustained, giving slightly reduced values at the second hour after administration. On the other hand, diabetic animals treated with HEPA showed a significant reduction at the first hour of treatment, and this effect remains during all experiment (Figure 4(b)). Some hypotheses may be raised to explain this amelioration observed in the oral glucose tolerance test, including lowered intestinal glucose absorption and improvement in the delayed insulin response. Accordingly, other workers have utilized the same experimental design to demonstrate the antidiabetic and glucose tolerance effects of plant extracts in experimentally induced diabetic rats [48–50].

Different mechanisms of action of herbal extracts have been reported to explain improvements in glycemic levels. Some plants present actions similar to the well-known sulfonylurea, which produces hypoglycemia in nondiabetic animals [51]. Some other plants act similarly to biguanides, an antihyperglycemic compound that act inhibiting the gluconeogenesis and do not affect blood glucose levels in normoglycemic animals [52]. Since *P. aculeata* did not reduce glycemia in normal rats, is possible that it acts in a similar way to biguanides. However, the capacity of *P. aculeata* to regenerate pancreatic *β*-cells should not be excluded as a mechanism of action.

## 5. Conclusion

**Figure 5:**
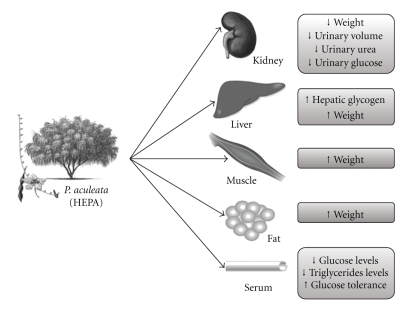
*Schematic representation of the proposed effects of the HEPA treatment in alloxan-induced diabetic rats. *The group of diabetic rats which were treated with the HEPA showed a significant reduction in serum and urinary glucose, urinary urea, and triglyceride levels. Also, an improvement in hepatic glycogen was observed as well as a decrease in liquid intake and urinary volume, and an enhancement in the weight of skeletal muscles, kidneys, liver, and adipose tissue. Indeed, oral glucose tolerance was higher in diabetic animals treated with HEPA. Together, all observed changes in some of the measured parameters, suggests a reduction on the gluconeogenesis process.

Based on this study, we conclude that the aerial parts of *P. aculeata* represent a good candidate for alternative and/or complementary medicine in the management of diabetes mellitus, since the plant extract treatment exhibited a high degree of toxicological safety and several beneficial effects on diabetic animals (see Figure 5). Additionally, the hypotriglyceridemic effect of HEPA, observed in normal rats, should be highlighted. As observed in our previous studies [13], the hydroethanolic extract of *P. aculeata* also did not show a dose-dependent effect on the majority of parameters studied. Finally, results also indicate that *P. aculeata* contains polar antidiabetic components.

## Figures and Tables

**Table 1:** Phytochemical result of the aerial parts hydroalcoholic extract of *Parkinsonia aculeata. *

Tests	Result
*Alkaloids*	
(i) Drangendoff's test	−
(ii) Wagner's test	−
(iii) Mayer's test	−
*Saponins*	
(i) Emulsion test	−
(ii) Frothing test	−
*Tanins*	
(i) Bromine water test	++
(ii) Ferric chloride test	++
*Flavonoids*	
(i) Sodium chloride test	+++
(ii) Magnesium ribbon test	+++
*Steroids*	
(i) Lieberman-Burchard test	++

(−) Not detected; (+) present in low concentration; (++) present in moderate concentration; (+++) present in high concentration.

**Table 2:** Serum total cholesterol, HDL-cholesterol, and triglycerides levels in normal and diabetic rats after subchronic treatment with HEPA.

Groups		Lipids concentration (mg/dL)	
	Total Cholesterol	HDL-cholesterol	Triglycerides
NC	77.34 ± 2.45	36.3 ± 1.5	103.27 ± 5.50
NT125	81.72 ± 2.47	40.6 ± 1.2	83.68 ± 3.93^#^
NT250	84.45 ± 5.83	41.2 ± 1.3	71.21 ± 4.18^#^
DC	85.83 ± 7.72	32.4 ± 2.2	253.92 ± 18.77
DT125	73.26 ± 9.74	33.9 ± 4.1	78.38 ± 10.52^∗a^
DT250	79.45 ± 3.92	37.7 ± 2.1	65.34 ± 8.68^∗a^
DTI	83.81 ± 3.43	41.2 ± 1.8*	132.15 ± 16.29^∗a^

Data represent the end of treatment (Day 16). NC: nondiabetic control (vehicle); NT125: nondiabetic treated with HEPA (125 mg/Kg); NT250: nondiabetic treated with HEPA (250 mg/Kg); DC: diabetic control (vehicle); DT125: diabetic treated with HEPA (125 mg/Kg); DT250: diabetic treated with HEPA (250 mg/Kg); DTI: diabetic treated with insulin. The values represent the mean ± S.E.M. (*n* = 6). One-way ANOVA with Tukey's *post hoc* test. *P < .05 versus DC group; ^#^ *P* < .05 versus NC group; ^a^ *P* < .05 versus DTI group.

**Table 3:** Effects of subchronic treatment with HEPA on body weight, physiological variables, and weights (tissues (g) per 100 g of body weight) of liver, kidneys, epididymal adipose tissue (EAT), *soleus *and *extensor digitorium longus* (EDL) in alloxan-diabetic rats.

Metabolic Parameters	NC	DC	DT125	DT250	DTI
Body Weight (g)	224.8 ± 6.4	147.8 ± 6.3	156.9 ± 10.2	172.7 ± 5.3*	205.5 ± 4.5*
	(+10.8%)	(−9.5%)	(−7.3%)	(+8.2%)	(+22.5%)
Liquid Intake (mL/24 h)	28.3 ± 1.6	193.8 ± 7.5	140.1 ± 8.3*	128.6 ± 6.2*	90.3 ± 5.9*
Food Intake (g/24 h)	18.2 ± 1.8	35.6 ± 0.8	32.8 ± 1.2	30.1 ± 1.8	28.5 ± 1.2
Urinary Volume (mL/24 h)	6.7 ± 1.1	136.3 ± 1.8	78.5 ± 4.1*	93.1 ± 3.5*	60.3 ± 4.7*
Liver	4.85 ± 0.25	6.04 ± 0.20	5.37 ± 0.35	5.07 ± 0.17*	5.29 ± 0.21*
Kidneys	0.44 ± 0.02	0.76 ± 0.02	0.49 ± 0.03^a^	0.47 ± 0.03^a^	0.46 ± 0.01^a^
EAT	0.87 ± 0.20	0.14 ± 0.01	0.19 ± 0.01*	0.95 ± 0.08*	0.79 ± 0.06*
*Soleus*	0.07 ± 0.00	0.05 ± 0.00	0.06 ± 0.00^#^	0.07 ± 0.00^#^	0.06 ± 0.00^#^
EDL	0.06 ± 0.00	0.04 ± 0.00	0.05 ± 0.00*	0.06 ± 0.00*	0.05 ± 0.00*

Data represent the end of treatment (Day 16). NC: nondiabetic control (vehicle); DC: diabetic control (vehicle); DT125: diabetic treated with HEPA (125 mg/Kg); DT250: diabetic treated with HEPA (250 mg/Kg); DTI: diabetic treated with insulin. Values given are the mean ± S.E.M. (*n* = 6). One-way ANOVA with Tukey's *post hoc* test. *P < .05; ^#^ *P* < .01; ^a^ *P* < .0001 versus DC. Percentage changes (figures in parenthesis) in body weight of all groups were in comparison with yours initial (Day 0—opening of treatment).
